# Congenital Peritoneal Encapsulation: A Literature Review of a Rare Pathology

**DOI:** 10.7759/cureus.31765

**Published:** 2022-11-21

**Authors:** Ashim Chowdhury, Ravi Chandra Tata, Ankur Shah, Veera Jaya Chandra Allu

**Affiliations:** 1 Department of General Surgery, William Harvey Hospital, Ashford, GBR

**Keywords:** surgery, rare congenital anomaly, small intestinal obstruction, abdominal cocoon, congenital peritoneal encapsulation

## Abstract

Congenital peritoneal encapsulation (CPE) is a rare, congenital entity in which an accessory peritoneal membrane surrounds the small bowel. This condition is usually asymptomatic and rarely causes intestinal obstruction. Despite the rare cause of intestinal obstruction, it has excellent post-operative recovery. There is no gold standard approach for investigating CPE; however, a computerized tomography scan of the abdomen might be helpful. Furthermore, diagnostic laparoscopy could be considered an adjunct. This report highlights the rare congenital anomaly as a cause of intestinal obstruction.

## Introduction and background

Congenital peritoneal encapsulation (CPE) is an extremely rare congenital malformation in which the small bowel is partially or totally surrounded by an accessory membrane [1]. Mostly, the condition remains asymptomatic and is an incidental diagnosis during surgery or autopsy. The condition was first reported by Cleland in 1868 as a “congenital anomaly that occurs secondary to an abnormal return of abdominal wall contents during the 12th week of gestation” [2]. Other rare entities causing small bowel encapsulation include abdominal cocoon (AC) and sclerosing encapsulated peritonitis (SEP), often mimicking CPE [3]. While used interchangeably, these are separate pathologies.

Being the rare cause of intestinal obstruction, CPE is often overlooked as a potential differential diagnosis for recurrent, undifferentiated abdominal pain and subacute small bowel obstruction. While it remains underdiagnosed, compounded by the difficulty in identification through imaging, patients treated with prompt surgical management have shown excellent postoperative recovery [1].

This report aims to highlight the rare congenital anomaly as a cause of intestinal obstruction and consolidate existing literature on CPE.

## Review

Methods

**Figure 1:**
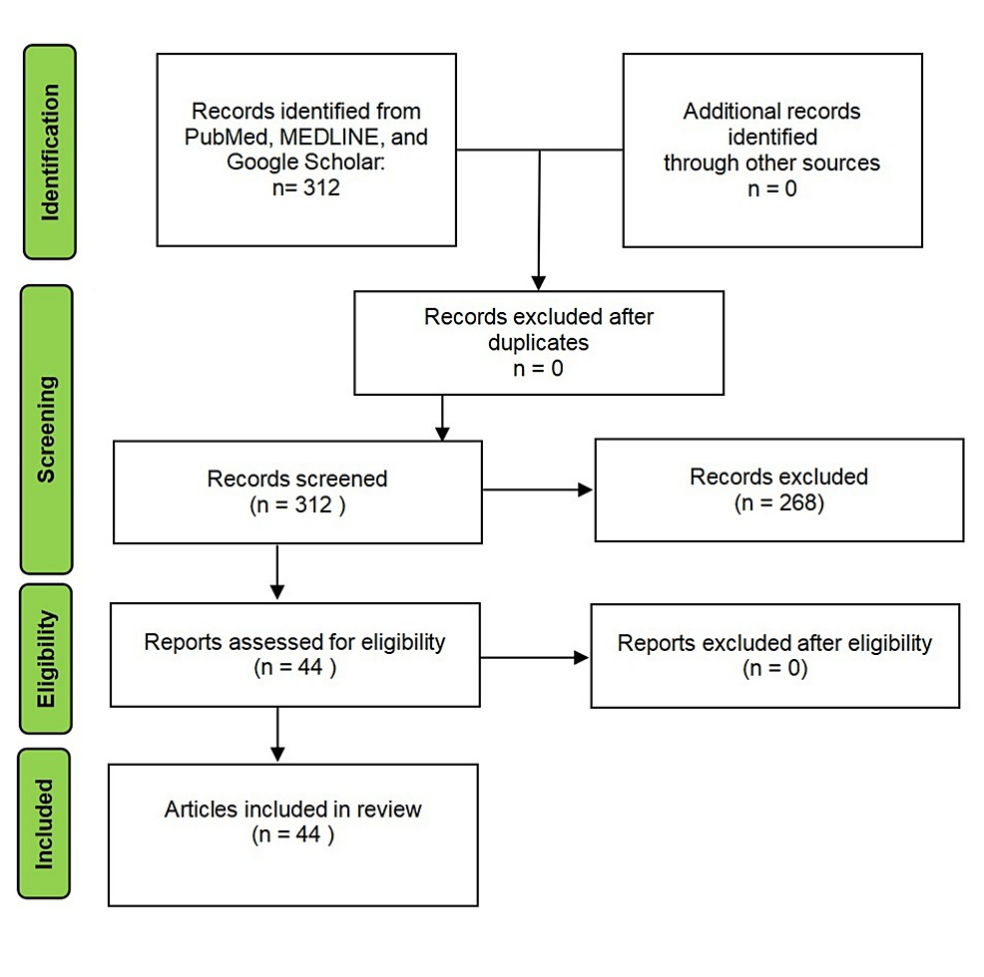
PRISMA flowchart PRISMA: Preferred Reporting Items for Systematic Reviews and Meta-Analyses

A systemic search of databases such as PubMed, Medline, and Google Scholar was carried out. The keywords to search for relevant articles were “abdominal cocoon, peritoneal encapsulation, and congenital peritoneal encapsulation.” The results yielded a total of 312 articles. The authors performed a manual screening of the articles. A higher number of these articles were related to SEP and excluded from this review. Where full-text articles were not available, abstracts with adequate information were included in this review (Figure 1).

Result

**Figure 2:**
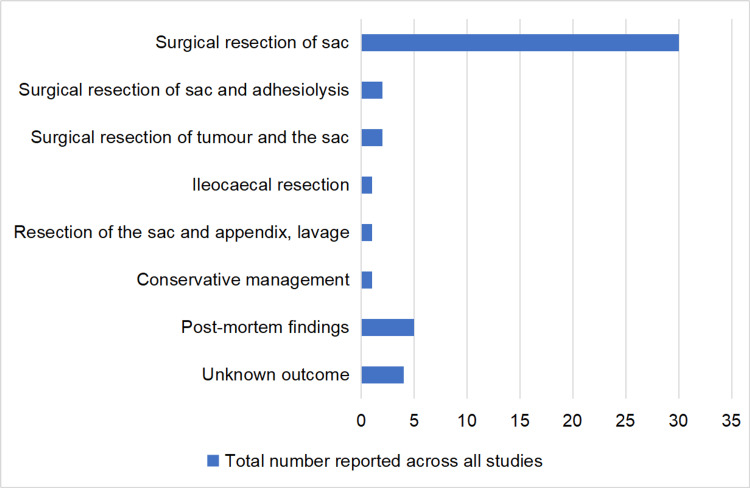
Interventions reported across all the studies

A total of 44 articles were finalized for this review [2-45]. The articles include predominantly case reports and four literature reviews. The oldest literature review included is from 1979, with the latest carried out by Dave et al. in 2019 [1]. The authors systematically screened the articles to extract demographic data, such as the age ranges of the patients, along with the presenting symptoms and radiological studies used in diagnoses. The most common age range was between 10 and 19 (Table 1); additionally, the cases reported also noted a male predominance (30 males and 15 females). The most common presenting complaint was abdominal pain followed by signs of bowel obstruction (Table 2). X-ray was the most common radiological investigation used as an adjunct in diagnosis (Table 3). The intervention used in most reported cases was surgical resection of the sac (Figure 2).

**Table 1:** Distribution of patients by age in published reports

Age (by decade)	Number of patients reported across all studies
10-19	10
20-29	9
30-39	2
40-49	6
50-59	5
60-69	6
70-79	2
80-90	3
Grand Total	43

**Table 2:** Distribution of presenting symptoms across published reports

Presenting symptoms	Number of patients reported across all studies
Abdominal Pain (Acute)	14
Signs of Small Bowel Obstruction	9
Incidental Finding	5
Abdominal Pain (Chronic)	8
Abdominal Pain (Acute) + Bilious Vomiting	1
Abdominal Pain (Acute) + Non-Bilious Vomiting	1
Groin Pain	1
Grand Total	39

**Table 3:** Radiologic investigations used across published studies CECT - Contrast-enhanced computed tomography

Investigations	Total number reported across all studies
Plain X-Ray	30
X-Ray With Contrast	4
Ultrasound	11
Computerized Tomography (Including CECT)	17

Discussion

CPE occurs due to a developmental anomaly resulting in an accessory intraperitoneal membrane, typically found between the mesocolon and the omentum, believed to be derived from the yolk sac peritoneum in early fetal development [46]. The accessory peritoneum is a transparent membrane formed by the peritoneal layer of mesothelial cells with few adhesions, usually encasing the entire small bowel. In one of the first few publications reporting the anomaly, Lickley et al. present a post-mortem report of a 52-year-old male, with a cause of death unrelated to intestinal pathology, as having ‘embryological importance [45]. They attribute the cause of the encapsulation to an abnormal gut rotation during embryological development, where the original mesentery is retained and the subsequently developing mesentery on the small intestine trapping the contents in a ‘peritoneal sac.’ Thorlakson et al. explain that the encapsulation results follow an abnormal reduction of physiological hernia [43]. However, Dave et al. report that this explanation does not counter the usual lack of gut-associated malformations [1].

In rare cases, as described by Casas et al., the membrane only encases some of the small bowel loops [28]. It occurs more frequently in young male patients and may be associated with intestinal malrotation [1,4]. The fixation points of this peritoneal sac are laterally the ascending and descending colon, the transverse colon superiorly, and the posterior parietal peritoneum inferiorly [2].

AC and CPE seem to be used interchangeably in the published literature. AC describes a fibrous membrane encasing the small bowel fully or partially, resulting in ‘cocooning.’ Bassiouny et al. make the case to distinguish AC from congenital encapsulation by calling the latter ‘small bowel cocoon’ [46]. Contrary to AC, CPE is primarily asymptomatic and discovered incidentally later in life, with only four cases reported in children. Dave et al. point out that SEP and AC form part of the differential diagnosis [1]. CPE differs from fibrotic encapsulating bowel diseases in that the latter usually have known secondary causes, from peritoneal dialysis, local irritation, peritoneal shunts, peritoneal tuberculosis, intraperitoneal chemotherapy, etc. [47-49]. Xu-Hua et al. report a case of CPE discovered incidentally during a radical resection of sigmoid colon cancer, noting the lack of infiltration of nodules in the small intestine and mesentery within the encapsulation [50]. This may point to the role of CPE having an unintentional benefit in patients with intrabdominal malignancies. Al-Taan et al. also report a case of incidental CPE finding during surgery for colon cancer in an 82-year-old man [18]. Of interest, Tsunoda et al. describe encountering SEP combined with peritoneal encapsulation, which is potentially the only reported case with such pathology [31].

Although a rare occurrence in CPE, most cases have reported presentation with a non-strangulating small intestinal obstruction. There are no identifying clinical features for diagnosis of CPE itself; however, Narayansingh et al. note that due to the fibrous encapsulation of the intestine, distension will be limited to the bowel proximal to and outside of it [3]. They note two clinical signs that can be observed - a fixed, asymmetrical abdominal distention not varying with peristalsis and a difference in consistency of the abdominal wall to palpation, where the distended area is soft and the flat area is firm [7]. However, it is important to note that these examination signs have only been noted in 10% of cases [1], and therefore relying on a clinical diagnosis may point to why these cases go underdiagnosed. Given that the majority of cases present with small bowel obstruction, the presenting clinical features will be akin to the symptoms and signs of small bowel obstruction, which include abdominal pain, tenderness on palpation, nausea, and vomiting. The main presenting symptom is abdominal pain, which was noted in 66% of patients [1]. The abdominal pain ranged from acute, chronic, and intermittent, which indicated that there is a role for the consideration of CPE as a differential for intermittent abdominal pain of no other obvious cause.

Radiology is not usually diagnostic, with radiography or ultrasonography showing no pathology on routine check-ups. As it is an incidental finding and usually benign, there could be inadequate reports on the radiological manifestations of CPE. Casas et al. note that diagnosis by CT is possible and describe a case where a CT performed on an asymptomatic patient showed the accessory peritoneum enveloping most of the ileal loops [28]. They note the similarities of the CT findings with AC and CPE and point out that the diagnosis can be differentiated clinically. Chew et al. report a 38-year-old man with intestinal obstruction, where the preoperative CT showed a localized fluid collection encasing the small intestine. Cases such as this could make diagnosis easier, as the fluid encapsulation highlights the presence of the capsule itself. However, it is unclear whether this led to a preoperative diagnosis, as the paper describes an attempt to manage conservatively [22].

**Figure 3:**
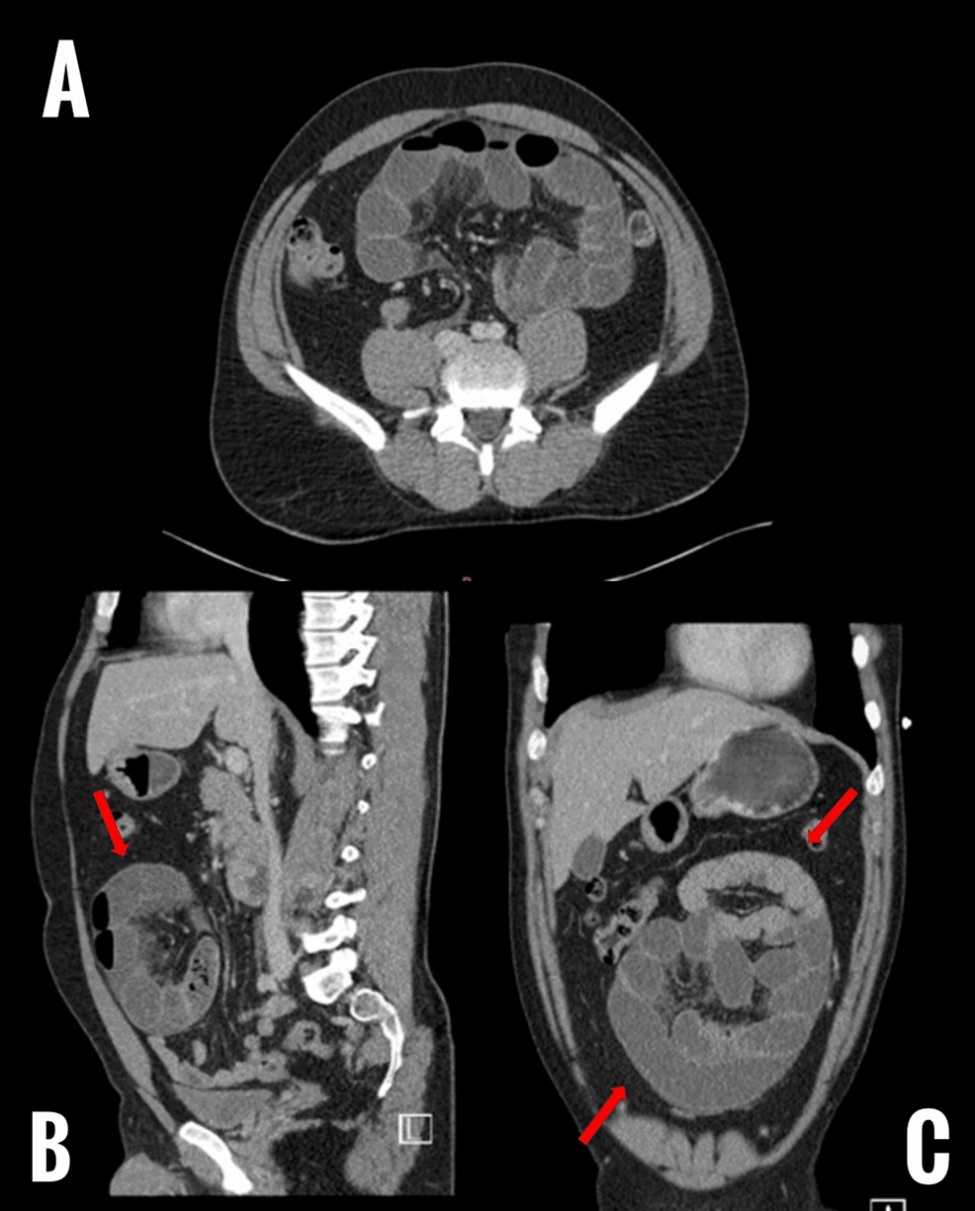
Computerized tomography scan images of our institutional case (A - Axial view, B - Sagittal view, C - Coronal view) Features of loculated, encysted, mechanical small-bowel obstruction with a sac-like appearance (red arrows) containing dilated jejunal and proximal iliac loops.

Silva MB Jr et al. report a case of a young asymptomatic individual that had rapid development of acute aortic occlusion secondary to CPE [32]. Specifically, the aortic occlusion resulted from external compression of the juxtarenal aorta from encapsulated closed-loop small intestinal obstruction. As such, they note that this could potentially mean that CPE might not always be a benign congenital anomaly with no role in emergencies. This could highlight a potential need for earlier diagnosis and treatment of the congenital abnormality. A series of CT images showing CPE can be seen here in Figure 3.

Dave et al. present a classification system based on histo-morphology to distinguish between CPE, SEP, and AC. They differentiate CPE from SEP and AC, as the latter two conditions seem to be acquired pathologies, the cause of which may be known such as abdominal trauma of various sources or idiopathy. SEP and AC are also seen to affect the small and large bowel and produce a thick fibro-collagenous capsule; which points to a different etiology as compared to CPE. They use the term fibrotic peritoneal encapsulation (FPE) to describe the later pathologies, allowing it to be separated from CPE [1]. FPE is attributed to inflammatory causes and results in scarring, eventually producing a fibro-collagenous capsule that can encapsulate both the small and large bowel. On the other hand, CPE is a rare congenital abnormality that produces a thin, semi-clear layer similar to the peritoneum. CPE and FPE also differ in the prognosis; where the prognosis for CPE is excellent with surgery, the prognosis for FPE can have a mortality of up to 50% within the first year after diagnosis.

Management of CPE can be divided into conservative, medical, and surgical management. However, given that most cases present with small bowel obstruction, treating conservatively is rare. Dave et al. refer to one case that was managed conservatively, as the CPE was detected incidentally and did not present with any pathological symptoms.

In terms of medical management, it is mainly centered around the management of small bowel obstruction with resuscitation to stabilize the patient and decompression using a nasogastric tube. On the other hand, surgery is curative, provided there is no ischaemic bowel. The surgical management is mainly centered around adhesiolysis and peritonectomy to remove this accessory sac [9], as was the case with the patient we had encountered. Given the straightforward nature of this procedure, the postoperative recovery is usually uneventful.

As CPE is an incidental finding during surgery, often to manage intestinal obstruction, intervention is often required as an emergency procedure. If needed, excision of the membrane and adhesiolysis between the loops is carried out with a high survival rate and low recurrence.

## Conclusions

Knowledge of CPE is essential for a surgeon as part of the differential diagnosis to avoid confusion during surgery, primarily via the laparoscopic approach. Notably, this diagnosis comes into account when encountering small bowel obstruction without other etiological factors. While imaging is not diagnostic, knowing what to expect in CPE can allow the surgeon to make a preoperative diagnosis based on collaborative findings during examination and etiology. A more extensive prospective study with a higher study population would be ideal for establishing a standard treatment protocol for CPE, a rare condition.
